# Quantifying and Adjusting for Confounding From Health-Seeking Behavior and Health Care Access in Observational Research

**DOI:** 10.1093/ofid/ofae598

**Published:** 2024-10-23

**Authors:** Sophie Graham, Jemma L Walker, Nick Andrews, William J Hulme, Dorothea Nitsch, Edward P K Parker, Helen I McDonald

**Affiliations:** Faculty of Epidemiology and Population Health, London School of Hygiene and Tropical Medicine, London, UK; Health Protection Research Unit in Vaccines and Immunisation, National Institute for Health and Care Research, London, UK; Faculty of Epidemiology and Population Health, London School of Hygiene and Tropical Medicine, London, UK; Health Protection Research Unit in Vaccines and Immunisation, National Institute for Health and Care Research, London, UK; Statistics Unit, UK Health Security Agency, London, UK; Health Protection Research Unit in Vaccines and Immunisation, National Institute for Health and Care Research, London, UK; Statistics Unit, UK Health Security Agency, London, UK; The Bennett Institute for Applied Data Science, Nuffield Department of Primary Care Health Sciences, University of Oxford, Oxford, UK; Faculty of Epidemiology and Population Health, London School of Hygiene and Tropical Medicine, London, UK; UK Renal Registry, Bristol, UK; Renal Unit, Royal Free London NHS Foundation Trust, Hertfordshire, UK; Faculty of Epidemiology and Population Health, London School of Hygiene and Tropical Medicine, London, UK; Health Protection Research Unit in Vaccines and Immunisation, National Institute for Health and Care Research, London, UK; Faculty of Epidemiology and Population Health, London School of Hygiene and Tropical Medicine, London, UK; Health Protection Research Unit in Vaccines and Immunisation, National Institute for Health and Care Research, London, UK; Statistics Unit, UK Health Security Agency, London, UK; Faculty of Science, University of Bath, Bath, UK

**Keywords:** confounding from health-seeking behavior and health care access, COVID-19, influenza, vaccine effectiveness, vaccines

## Abstract

**Background:**

Health-seeking behavior and health care access (HSB/HCA) are recognized confounders in many observational studies but are not directly measurable in electronic health records. We used proxy markers of HSB/HCA to quantify and adjust for confounding in observational studies of influenza and COVID-19 vaccine effectiveness (VE).

**Methods:**

This cohort study used primary care data prelinked to secondary care and death data in England. We included individuals aged ≥66 years on 1 September 2019 and assessed influenza VE in the 2019–2020 season and early COVID-19 VE (December 2020–March 2021). VE was estimated with sequential adjustment for demographics, comorbidities, and 14 markers of HSB/HCA. Influenza vaccination in the 2019–2020 season was also considered a negative control exposure against COVID-19 before COVID-19 vaccine rollout.

**Results:**

We included 1 991 284, 1 796 667, and 1 946 943 individuals in the influenza, COVID-19, and negative control exposure populations, respectively. Markers of HSB/HCA were positively correlated with influenza and COVID-19 vaccine uptake. For influenza, adjusting for HSB/HCA markers in addition to demographics and comorbidities increased VE against influenza-like illness from −1.5% (95% CI, −3.2% to .1%) to 7.1% (95% CI, 5.4%–8.7%) with a less apparent trend for more severe outcomes. For COVID-19, adjusting for HSB/HCA markers did not change VE estimates against infection or severe disease (eg, 2 doses of BNT162b2 against infection: 82.8% [95% CI, 78.4%–86.3%] to 83.1% [95% CI, 78.7%–86.5%]). Adjusting for HSB/HCA markers removed bias in the negative control exposure analysis (−7.5% [95% CI, −10.6% to −4.5%] vs −2.1% [95% CI, −6.0% to 1.7%] before vs after adjusting for HSB/HCA markers).

**Conclusions:**

Markers of HSB/HCA can be used to quantify and account for confounding in observational vaccine studies.

Health-seeking behavior and health care access may be important confounders in observational research. Health-seeking behavior is defined as seeking care for disease prevention when asymptomatic or during early symptomatic stages [1], and health care access is defined as the ability to access health care services for these purposes [2]. Individuals with active health-seeking behavior and the ability to easily access health care services generally have favorable clinical outcomes [3]. Confounding from health-seeking behavior and health care access has led to overestimates of effectiveness of preventative therapies in observational research. For example, observational studies of statin use have consistently shown a reduction in hip fracture risk, even though this is not reflected in clinical trials [4]. In cohort studies of influenza vaccine effectiveness (VE), authors have reported reductions in all-cause mortality by 40% to 50% [5, 6], despite influenza accounting for a maximum of 10% of deaths per year [7]. However, systematically accounting for this type of confounding is challenging, as health-seeking behavior and health care access are not directly measurable in routine health care data.

Proxy markers identified in electronic health records (EHRs) have been used to attempt to account for confounding from health-seeking behavior and health care access [8–12]. Markers vary considerably and optimal approaches are unclear. We recently identified a systematic set of 14 markers of health-seeking behavior and health care access in UK EHRs [13] that accounted for a range of determinants based on the “theory of planned behavior” model [14]. These markers represent health care system interactions that are only partially driven by an individual's underlying health need. In the current study, we aimed to assess whether these proxy markers of health-seeking behavior and health care access can be used to quantify and adjust for confounding in observational studies, using seasonal influenza and COVID-19 VE as examples.

## METHODS

### Data Sources

We conducted a cohort study using Clinical Practice Research Datalink (CPRD) Aurum [15] prelinked to Hospital Episode Statistics Admitted Patient Care [16] and Office for National Statistics data [17]. CPRD Aurum includes diagnoses and other medical terms recorded by SNOMED, Read Coded Clinical Terms version 3, or local EMIS codes. Each internally mapped to an individual “medcode,” and we use this term for brevity. CPRD Aurum also comprises the primary care prescriptions (recorded per the Dictionary of Medicines and Devices codes [18]) and referral and testing information of patients registered to consenting general practitioner (GP) practices in the United Kingdom. Hospital Episode Statistics Admitted Patient Care consists of all admissions to National Health Service (NHS) hospitals in England [16]. It includes inpatient hospital admission and discharge dates, diagnoses recorded per *ICD-10* revision codes [19], and procedures recorded per Operating Procedure Codes Supplement codes [20]. The Office for National Statistics comprises the date and underlying cause of death, recorded per *ICD-10* codes, and socioeconomic data based on the index of multiple deprivation (IMD) [21], which is based on small area geographic location. At the time of data extraction, CPRD Aurum consisted of 13 300 067 currently contributing patients (19.8% of the UK population) [22].

### Study Design and Population Selection

We created separate cohorts to estimate influenza and COVID-19 VE (Supplementary Table 1). In addition, to assess potential residual confounding, we created a third “negative control exposure” cohort. Negative control exposures assume no causal mechanism between the negative control exposure and outcome, and they include confounding structures that reflect those of the primary exposure [23]. We used 2019–2020 seasonal influenza vaccinations as a negative control exposure against COVID-19 infections before COVID-19 vaccinations were available in the United Kingdom.

**Figure 1.**
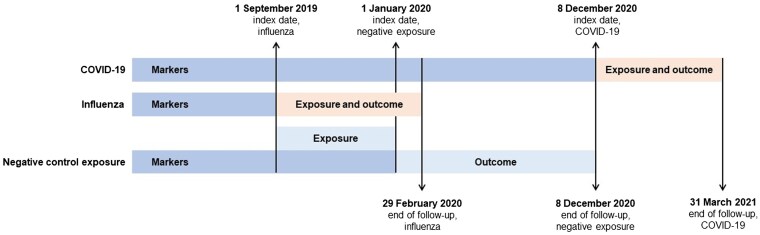
Study design. We followed individuals until the earliest of death, transfer out of general practitioner practice, end of data availability (COVID-19), end of influenza season (influenza), or start of COVID-19 vaccination rollout (negative control exposure). For the COVID-19 cohort, we censored individuals at first COVID-19 vaccination that was neither BNT162b2 nor ChAdOx1 or on receipt of a second heterologous vaccination.

We included all persons aged ≥66 years (on 1 September 2019), who are prioritized for both vaccines and likely to show distinct patterns of health-seeking behavior and health care access [24]. The index date was 1 September 2019 for the influenza cohort, 8 December 2020 for the COVID-19 cohort, and 1 January 2020 for the negative control exposure cohort. To create the separate cohorts, we required all individuals to have at least 1 year of registration prior to their index date, a record of “acceptable” quality by CPRD (see Supplementary Information), and linkage eligibility to Hospital Episode Statistics Admitted Patient Care and the Office for National Statistics. We excluded those with a death or registration end date before the index or with indeterminate sex (n = 8). Patients in the COVID-19 cohort were additionally excluded if their first vaccination was prior to 8 December 2020, as these likely reflected coding errors or trial participants (Figure 1, Supplementary Table 1).

### Outcomes, Exposures, and Follow-up

For all analyses, we considered 3 nested outcomes of increasing severity: cause-specific infections (identified in primary care as a hospitalization or death), hospitalizations/deaths, and deaths. For influenza, as influenza testing is infrequently conducted in the United Kingdom, we required a cause-specific diagnosis of acute respiratory infection or influenza-like illness (ARI/ILI) [25]. For all hospital and death outcomes, the diagnosis code was required to be in the primary position.

We identified BNT162b2 and ChAdOx1 COVID-19 vaccines separately and requiring a minimum interval of 18 days between first and second doses [26]. We identified COVID-19 vaccinations using prescription records automatically recorded in GP records. For influenza, we identified vaccinations in the 2019–2020 season using medcodes and prescription records using an algorithm (Supplementary Table 2).

For influenza, the index date was 1 September 2019, and individuals were followed up until the earliest of death, transfer out of the practice, or start of the COVID-19 pandemic (29 February 2020). For COVID-19, the index date was 8 December 2020, when COVID-19 vaccinations were introduced in the United Kingdom. Patients were followed up until the earliest of death, transfer out of the practice, end of data availability (31 March 2021), first vaccination that was neither BNT162b2 nor ChAdOx1, or second heterologous vaccination. For the negative control exposure analysis, the index date was 1 January 2020, when the first SARS-CoV-2 infections were identified in the United Kingdom. We included influenza vaccinations before 31 December 2019, by which time the majority of vaccinations in the United Kingdom had been delivered to reflect positive health-seeking behavior/access and prevent overlap with the outcome period. Patients were followed up until the earliest of death, transfer out of the practice, or the day before introduction of COVID-19 vaccinations in the United Kingdom (7 December 2020).

### Sociodemographic Variables

At the index for each cohort, we described the following: age (based on year of birth), sex, recent infection (<3 months preindex for SARS-CoV-2 or within the previous season for influenza), IMD, ethnicity [27], and influenza “at risk” conditions [28]. Influenza “at risk” groups [28] were identified from primary care records, as described previously [25], and grouped into immunosuppression or other conditions. We assessed missingness of ethnicity, IMD, and region. For all other variables, absent codes were regarded as evidence of absence.

### Markers of Health-Seeking Behavior and Health Care Access

We used 14 markers of health-seeking behavior and health care access that represented (1) uptake of public health interventions (abdominal aortic aneurysm, breast cancer, cervical cancer, and bowel cancer screening; influenza and pneumococcal vaccinations and NHS health checks), (2) active health care access/use (prostate-specific antigen testing, bone density scans, primary care visits, low value procedures [29], and blood pressure measurements), and (3) lack of access/underuse (hospital visits for ambulatory care–sensitive conditions [30] and “did not attend” primary care visits). These were all identified previously [13] through a framework based on the updated “theory of planned behavior” model [14]. Markers were identified in primary care and hospital records as described previously [13]. The lookback periods reflect use of these resources in UK clinical practice (Supplementary Table 1 has details on all variable definitions).

### Statistical Analyses

We described sociodemographic variables, clinical variables, and markers of health-seeking behavior at the index, stratified by final vaccination status. To assess timeliness of vaccination, we calculated median days from the index to first vaccination among vaccinated individuals, stratified by marker status and age categories (to reflect UK COVID-19 vaccination phased deployment [9]). Outcome rates were represented by vaccination status as the number of events divided by total person-years. We used Cox regression models to estimate outcome risk in vaccinated vs unvaccinated persons. A complete case analysis was conducted (excluding those with missing region, ethnicity, or IMD). In the influenza and COVID-19 analyses, vaccination status was time updated, with all individuals starting follow-up unvaccinated and with vaccination status updated 14 days after a vaccination date (to provide time for an immune response). For COVID-19, the analysis was brand specific. We assessed VE as (1 – hazard ratio) × 100.

We adapted a hierarchical modeling strategy [31] to understand the relationships among determinants of vaccine uptake in 4 steps. First, we fitted minimally adjusted models adjusting for age (quadratic polynomial), sex, region, and recent infection. Demography-adjusted models adjusted for ethnicity and IMD. Comorbidity-adjusted models adjusted for immunosuppressive status and other comorbidities. The fully adjusted models adjusted for health-seeking markers. For sex-specific markers (cervical cancer screening, breast cancer screening, abdominal aortic aneurysm screening, and prostate-specific antigen test), we included an interaction term with sex to allow us to generate 1 model that included both sexes to be generalizable to the overall English population.

We conducted a sensitivity analysis fitting age interactions with abdominal aortic aneurysm screening, bowel cancer screening, NHS health checks, and ambulatory care–sensitive conditions (all of which vary markedly by age) [13].

## RESULTS

### Study Population

**Figure 2.**
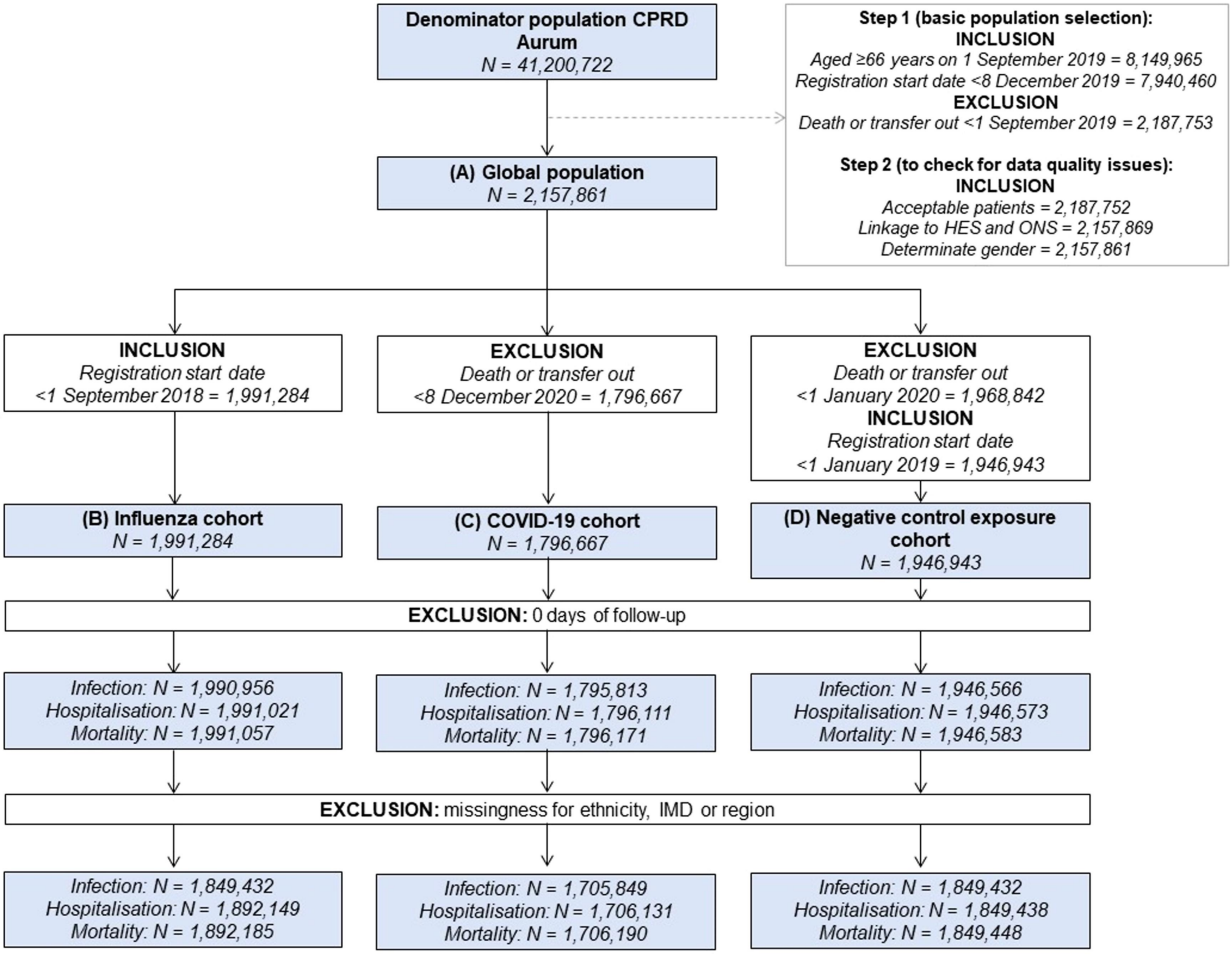
Study selection criteria. Additional details on population selection can be found in Supplementary Table 1. CPRD, Clinical Practice Research Datalink; HES, Hospital Episode Statistics; IMD, index of multiple deprivation; ONS, Office for National Statistics.

We included 1 946 943, 1 796 667, and 1 991 284 persons in the influenza, COVID-19, and negative control exposure cohorts, respectively (Figure 2). When compared with those who remained unvaccinated, vaccinated individuals were more likely to be older and of White ethnicity and to live in less deprived areas (Table 1, Supplementary Table 3).

**Table 1.** Baseline Characteristics Stratified by Vaccination Status at the End of Follow-up

	Influenza Analysis Population (n = 1 796 667)		COVID-19 Analysis Population (n = 1 796 667)			Negative Control Exposure Analysis Population (n = 1 946 943)	
Variable: Category	Vaccinated (n = 1 473 955)	Unvaccinated (n = 517 329)	ChAdOx1-S (n = 845 428)	BNT162b2 (n = 811 740)	Unvaccinated (n = 139 499)	Vaccinated (n = 1 437 356)	Unvaccinated (n = 509 587)
Age, y							
65–69	295 808 (20.1)	152 255 (29.4)	196 994 (23.3)	93 761 (11.6)	29 203 (20.9)	288 285 (20.1)	154 638 (30.3)
70–74	409 473 (27.8)	148 126 (28.6)	318 400 (37.7)	185 076 (22.8)	37 479 (26.9)	400 663 (27.9)	149 441 (29.3)
75–79	312 214 (21.2)	89 076 (17.2)	186 454 (22.1)	180 327 (22.2)	24 518 (17.6)	305 940 (21.3)	88 128 (17.3)
80–84	235 026 (15.9)	60 466 (11.7)	65 793 (7.8)	191 890 (23.6)	21 093 (15.1)	229 632 (16.0)	58 105 (11.4)
85–89	142 171 (9.6)	39 664 (7.7)	44 040 (5.2)	110 191 (13.6)	15 032 (10.8)	137 602 (9.6)	36 230 (7.1)
90–95	60 924 (4.1)	20 019 (3.9)	24 562 (2.9)	41 348 (5.1)	8520 (6.1)	58 241 (4.1)	17 037 (3.3)
>95	18 339 (1.2)	7723 (1.5)	9185 (1.1)	9147 (1.1)	3654 (2.6)	16 993 (1.2)	6008 (1.2)
Sex							
Female	795 391 (54.0)	280 332 (54.2)	458 466 (54.2)	442 085 (54.5)	73 243 (52.5)	776 347 (54.0)	276 181 (54.2)
Male	678 564 (46.0)	236 997 (45.8)	386 962 (45.8)	369 655 (45.5)	66 256 (47.5)	661 009 (46.0)	233 406 (45.8)
Ethnicity^a^							
Asian	49 874 (3.4)	18 087 (3.5)	27 362 (3.2)	26 576 (3.3)	9891 (7.1)	48 257 (3.4)	19 013 (3.7)
Black	21 970 (1.5)	14 942 (2.9)	12 665 (1.5)	9685 (1.2)	11 631 (8.3)	21 023 (1.5)	15 431 (3.0)
Mixed	6321 (0.4)	3751 (0.7)	3744 (0.4)	3223 (0.4)	2242 (1.6)	6095 (0.4)	3853 (0.8)
Other	11 295 (0.8)	6416 (1.2)	6750 (0.8)	5942 (0.7)	3841 (2.8)	10 982 (0.8)	6611 (1.3)
White	1 331 686 (90.3)	428 068 (82.7)	754 194 (89.2)	734 394 (90.5)	94 638 (67.8)	1 299 673 (90.4)	418 902 (82.2)
Missing	52 809 (3.6)	46 065 (8.9)	40 713 (4.8)	31 920 (3.9)	17 256 (12.4)	51 326 (3.6)	45 777 (9.0)
Region							
East Midlands	30 432 (2.1)	[Redacted]	18 636 (2.2)	14 776 (1.8)	1892 (1.4)	29 726 (2.1)	[Redacted]
East of England	70 073 (4.8)	25 340 (4.9)	39 048 (4.6)	37 023 (4.6)	5426 (3.9)	67 180 (4.7)	21 300 (4.2)
London	179 896 (12.2)	83 929 (16.2)	86 705 (10.3)	115 643 (14.2)	37 022 (26.5)	174 002 (12.1)	86 226 (16.9)
North East	51 140 (3.5)	16 138 (3.1)	30 256 (3.6)	28 324 (3.5)	3873 (2.8)	50 011 (3.5)	16 579 (3.3)
North West	288 382 (19.6)	93 210 (18.0)	165 164 (19.5)	158 595 (19.5)	23 774 (17.0)	282 152 (19.6)	93 857 (18.4)
South East	330 361 (22.4)	108 046 (20.9)	192 498 (22.8)	175 053 (21.6)	27 873 (20.0)	323 518 (22.5)	109 375 (21.5)
South West	202 112 (13.7)	68 372 (13.2)	126 645 (15.0)	103 675 (12.8)	14 764 (10.6)	196 664 (13.7)	62 755 (12.3)
West Midlands	262 952 (17.8)	94 411 (18.2)	151 012 (17.9)	151 300 (18.6)	21 235 (15.2)	256 602 (17.9)	94 795 (18.6)
Yorkshire and the Humber	58 599 (4.0)	16 206 (3.1)	35 386 (4.2)	27 336 (3.4)	3630 (2.6)	57 467 (4.0)	16 393 (3.2)
Unknown	8 (0.0)	[Redacted]	78 (0.0)	15 (0.0)	10 (0.0)	34 (0.0)	[Redacted]
IMD^b^							
1 (least deprived)	389 007 (26.4)	110 866 (21.4)	219 539 (26.0)	213 479 (26.3)	25 049 (18.0)	381 652 (26.6)	110 457 (21.7)
2	352 561 (23.9)	115 220 (22.3)	198 707 (23.5)	195 168 (24.0)	26 339 (18.9)	344 778 (24.0)	113 011 (22.2)
3	291 822 (19.8)	107 489 (20.8)	165 923 (19.6)	164 259 (20.2)	27 283 (19.6)	283 270 (19.7)	103 275 (20.3)
4	245 959 (16.7)	99 005 (19.1)	143 497 (17.0)	136 591 (16.8)	30 900 (22.2)	239 223 (16.6)	97 624 (19.2)
5 (most deprived)	194 606 (13.2)	84 749 (16.4)	117 762 (13.9)	102 243 (12.6)	29 928 (21.5)	188 433 (13.1)	85 220 (16.7)
Influenza “at risk” conditions							
Immunosuppressed status	44 445 (3.0)	10 708 (2.1)	20 560 (2.4)	20 314 (2.5)	3181 (2.3)	51 304 (3.6)	11 491 (2.3)
Other comorbidities^c^	875 433 (59.4)	227 851 (44.0)	455 435 (53.9)	478 945 (59.0)	70 209 (50.3)	859 858 (59.8)	224 804 (44.1)
Markers of health-seeking behavior and health care access							
AAA screen	171 329 (11.6)	59 759 (11.6)	76 400 (9.4)	127 455 (15.1)	11 087 (7.9)	167 605 (11.7)	59 711 (11.7)
Bowel screen	1 063 252 (72.1)	376 160 (72.7)	556 813 (68.6)	703 450 (83.2)	94 562 (67.8)	1 043 974 (72.6)	380 264 (74.6)
Breast screen	268 370 (18.2)	77 746 (15.0)	149 098 (18.4)	161 443 (19.1)	16 564 (11.9)	265 104 (18.4)	78 616 (15.4)
Cervical screen	308 261 (20.9)	89 042 (17.2)	171 781 (21.2)	171 059 (20.2)	20 103 (14.4)	301 116 (20.9)	87 878 (17.2)
NHS health checks	278 539 (18.9)	93 705 (18.1)	134 883 (16.6)	170 284 (20.1)	15 862 (11.4)	270 812 (18.8)	92 865 (18.2)
Influenza vaccine^d^	1 343 562 (91.2)	116 829 (22.6)	665 364 (82.0)	641 842 (75.9)	56 223 (40.3)	1 314 908 (91.5)	112 149 (22.0)
Pneumococcal vaccine	1 071 867 (72.7)	170 492 (33.0)	575 351 (70.9)	523 544 (61.9)	59 781 (42.9)	1 069 249 (74.4)	164 248 (32.2)
ACS hospital care visit	146 447 (9.9)	43 689 (8.4)	85 734 (10.6)	87 757 (10.4)	17 314 (12.4)	153 108 (10.7)	42 755 (8.4)
Blood pressure test	1 160 045 (78.7)	309 961 (59.9)	718 511 (88.5)	717 133 (84.8)	91 327 (65.5)	1 229 509 (85.5)	334 066 (65.6)
Bone density scan	81 881 (5.6)	19 011 (3.7)	56 679 (7.0)	53 276 (6.3)	5282 (3.8)	87 873 (6.1)	20 667 (4.1)
DNA primary care visit	459 941 (31.2)	141 955 (27.4)	431 120 (53.1)	432 191 (51.1)	72 675 (52.1)	560 407 (39.0)	171 514 (33.7)
Primary care visit	1 429 058 (97.0)	415 765 (80.4)	802 423 (98.9)	828 713 (98.0)	110 333 (79.1)	1 420 864 (98.9)	426 546 (83.7)
Low value procedures	280 546 (19.0)	78 335 (15.1)	259 273 (31.9)	243 222 (28.8)	34 634 (24.8)	342 882 (23.9)	93 058 (18.3)
PSA test	285 156 (19.3)	67 116 (13.0)	178 655 (22.0)	168 574 (19.9)	19 411 (13.9)	294 032 (20.5)	69 005 (13.5)

Data are presented as No. (%). For baseline characteristics in overall analysis populations, see Supplementary Table 3. For each analysis, we are comparing individuals with ≥1 vaccination vs no vaccination throughout follow-up. Cells with <5 individuals are redacted due to CPRD's patient confidentiality requirements, and secondary suppression has occurred where necessary. Age was estimated at the index date for each cohort, and since only year of birth is provided in the CPRD, all dates of birth were imputed as middle of the year (1 July).

Abbreviations: AAA, abdominal aortic aneurysm; ACS, ambulatory care sensitive; APC, Admitted Patient Care; CPRD, Clinical Practice Research Datalink; DNA, did not attend; HES, Hospital Episode Statistics; IMD, index of multiple deprivation; ONS, Office for National Statistics; PSA, prostate-specific antigen; VE, vaccine effectiveness.

^a^Ethnicity was identified from primary care records as described by Mathur et al [27]. Briefly, the algorithm uses a modal approach with ties resolved by recency. If ethnicity could not be identified in primary care, then ethnicity from HES APC was used.

^b^IMD was identified from the ONS at the patient level or, if missing, by the primary care practice.

^c^Other comorbidities: chronic liver disease, chronic cardiac disease, chronic respiratory disease, asthma, diabetes mellitus, chronic neurologic disease, chronic kidney disease, severe obesity, severe mental conditions, and severe learning disability. For more information on how these were defined, see Supplementary Table 1.

^d^Influenza vaccination that occurred in the influenza season prior to the index date. For COVID-19, this was an influenza vaccination that occurred 1 September 2019 to 31 March 2020; for influenza and negative control exposure, this was an influenza vaccination that occurred 1 September 2018 to 31 March 2019 and for negative control exposure that occurred from 1 September 2019 to 31 December 2019.

### Markers of Health-Seeking Behavior

When compared with those who remained unvaccinated, vaccinated individuals had a higher prevalence of all health-seeking markers (except ambulatory care–sensitive hospital visits, which should be prevented by health care access; Table 1). Differences in previous vaccinations were particularly marked. In the influenza analysis, 91.2% of vaccinated persons had an influenza vaccination in the previous season vs 22.6% of unvaccinated individuals for influenza vaccination, and a similar pattern was seen in COVID-19 analysis. Among vaccinated persons, time to vaccination was not strongly associated with health-seeking marker status, except previous season influenza vaccination, which was associated with faster uptake of COVID-19 and influenza vaccines (Supplementary Figures 1 and 2, Supplementary Tables 4 and 5).

### VE Estimates

**Figure 3.**
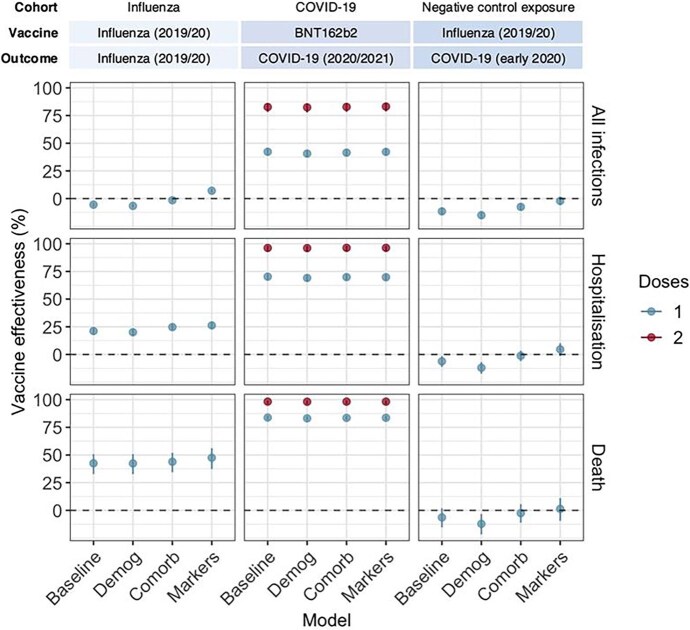
Estimated vaccine effectiveness following sequential confounder adjustment in each study analysis (columns) for each outcome of interest (rows). COVID-19 estimates are only for BNT162b2 vs unvaccinated, as ChAdOx1 follow-up data after 2 doses were limited. Baseline models adjusted for polynomial age, sex, region, and recent infection. The demography model was adjusted for ethnicity and index of multiple deprivation. Comorbidity models were adjusted for immunosuppressed status and other comorbidities. Marker models were adjusted for markers of health-seeking behavior and health care access. Data are presented as percentage vaccine effectiveness (95% CI). Comorb, comorbidities; demog, demography.

For influenza, the median (IQR) follow-up time was 181 (0) days, which included 50 (28) days after the first influenza vaccination. Unadjusted event rates ranged from 0.84 ARI/ILI-related deaths per 1000 person-years during the unvaccinated time to 117.15 ILIs per 1000 person-years after vaccination (Supplementary Table 6). Incremental adjustments across the models led to increased VE estimates. For ILI, we observed a negative VE in the minimally adjusted model (adjusting for age, sex, region, and recent infection) of −5.5% (95% CI, −7.2% to −3.9%). Estimated VE increased to −1.5% (95% CI, −3.2% to .1%) after additionally adjusting for demography (ethnicity and IMD) and comorbidities and to 7.1% (95% CI, 5.4%–8.7%) after additionally adjusting for health-seeking markers. For severe outcomes, estimated VE increased from 42.5% (95% CI, 32.8%–50.8%) against ARI/ILI-related death in the minimally adjusted model to 47.5% (95% CI, 37.3%–56.1%) in the fully adjusted model (Figure 3, Supplementary Table 7).

For COVID-19, the median (IQR) follow-up time was 113 (0) days, which included 64 (19) days after first BNT162b2 vaccination. Unadjusted event rates ranged from 0.54 COVID-19–related deaths per 1000 person-years after 2 doses of BNT162b2 vaccination to 95.39 COVID-19 infections per 1000 person-years during unvaccinated time (Supplementary Table 6). There was minimal change in VE from the minimally adjusted model to the fully adjusted model that included all health-seeking markers (eg, 2-dose VE against infection of 82.7% [95% CI, 78.3%–86.2%] and 83.1% [95% CI, 78.7%–86.5%], respectively). This was also the case for more severe outcomes (eg, 2-dose VE against hospitalization of 96.2% [95% CI, 93.0%–98.0%] and 92.3% [95% CI, 93.0%–98.0%] for minimally and fully adjusted models, respectively). For ChAdOx1, there was limited follow-up time after 2 doses (Supplementary Table 7).

For the negative control exposure analysis, the median follow-up time was 341 (0) days for those vaccinated and unvaccinated. Unadjusted event rates ranged from 1.39 COVID-19–related deaths per 1000 person-years for unvaccinated individuals to 13.77 COVID-19–related infections per 1000 person-years for influenza-vaccinated ones (Supplementary Table 6). We observed a negative VE for the effect of influenza vaccinations against COVID-19 for all minimally and demography-adjusted models (eg, −6.4% [95% CI, −15.4% to −1.9%] and −12% [95% CI, −17.4% to −6.9%], respectively, against COVID-19–related mortality). For infections, negative VE persisted after additionally adjusting for comorbidities (−7.5% [95% CI, −10.6% to −4.5%]) but not after additionally adjusting for health-seeking markers in the fully adjusted model (−2.1% [95% CI, −6.0% to 1.7%]). For more severe end points, additional adjustment for comorbidities led to VE estimates consistent with a null finding, which was also the case after additional adjustment for health-seeking markers (Figure 3, Supplementary Table 7).

Sensitivity analyses including interaction terms between age and age-varying markers did not substantively change VE estimates (Supplementary Table 7).

## DISCUSSION

Using a range of markers of health-seeking behavior, we were able to address confounding in VE studies of influenza and COVID-19, with a negative control exposure analysis demonstrating successful control of confounding. This was assessed via a large cohort of individuals aged ≥66 years in England, and confounding from health-seeking behavior and health care access was adjusted for by using proxy markers identified in UK EHRs. We found that influenza and COVID-19 vaccination uptake was higher in those with active health-seeking behavior and better health care access. For VE the impact of health-seeking behavior and health care access varied by context. Before the COVID-19 pandemic, VE against ILI was underestimated when health-seeking behavior was not adjusted for. This confounding was less apparent for more severe disease end points. For COVID-19 VE during a pandemic (during the early stages of COVID-19 vaccine implementation), minimally adjusted models were very similar to fully adjusted models that accounted for health-seeking behavior. Residual confounding was initially present and successfully removed by adjusting for health-seeking behavior in a negative control analysis of prepandemic influenza VE against early pandemic SARS-CoV-2 infections.

VE estimates from the comorbidity-adjusted models were similar to previous observational estimates. For influenza, a test-negative design study in the 2019–2020 season adjusted for age, sex, region, comorbidities, and calendar time in their logistic regression models and estimated VE against virology-confirmed disease to be 22.7% (95% CI, −38.5% to 56.9%) [32]. This is consistent with our estimate against ARI/ILI-related hospitalization or death (24.7% [95% CI, 22.0%–27.4%]). The low influenza VE estimate in our study (fully adjusted model, 7.1% [95% CI, 5.4%–8.7%]) may also be partly explained by underascertainment of infections and the nonspecific outcome of “influenza-like illness” that we used, rather than influenza, due to the lack of widespread testing for influenza in primary care settings and the subsequent nonspecific influenza coding. For COVID-19, a cohort study from December 2020 to April 2021 estimated VE among persons aged ≥65 years and adjusted for age, sex, ethnicity, IMD, prior COVID-19, large household, GP consultation quartile, comorbidities, shielding recommendation, and smoking status in Poisson regression models. The authors estimated VE as 84.7% (95% CI, 77.7%–89.5%) after 2 doses of BNT162b2 [12]—consistent with our all-infection estimate of 82.8% (95% CI, 78.4%–86.3%).

Our results differ from 2 US Medicare studies that assessed adjustment for proxy markers of health-seeking behavior and health care access on influenza and shingles VE estimates [33, 34]. Both studies saw a decrease in VE after adjusting for confounding from health-seeking behavior, whereas we saw an increase. These discrepancies could be due to differences in health care settings or data set types. The US studies [33, 34] also used a smaller set of markers; therefore, some residual confounding may have remained. One of the US studies [34] used preseason influenza estimates as a negative control outcome for influenza VE and found significant residual confounding in its fully adjusted model (32% [95% CI, 30%–33%]).

In the negative control analysis, we assumed that any plausible causal association between influenza vaccination and COVID-19 infection was minimal. Some studies with nonspecific COVID-19 outcomes have shown there to be a minor protective effect of the influenza vaccination against COVID-19 infection [35]. A recent observational study conducted with administrative data in Canada also reported a protective effect of influenza vaccinations against COVID-19 infections; however, it reported the same trend for previous health examination against COVID-19 infections (adjusted hazard ratio, 0.85 [95% CI, .78–.91]) [36]. The authors concluded that this provided evidence of residual confounding. Our study also identified and successfully removed residual confounding after adjusting for the health-seeking markers.

Future researchers will be able to use these markers to characterize health behaviors, to identify the strength and direction of confounding from health-seeking behavior and health care access, and to account for identified confounding. We believe that, particularly for seasonal influenza and COVID-19, these markers could be helpful to provide more accurate annual VE and cost-effectiveness estimates. They might also be important for chronic conditions (eg, chronic kidney disease and diabetes), for which health-seeking behavior and health care access have been shown to influence timeliness of seeking care and self-management [37, 38].

Usefulness of these markers is likely to vary by context. For example, they are likely to be more useful for routine rather than pandemic VE estimates. As we saw for COVID-19 during the pandemic, sequential model adjustments had limited impact on VE estimates. This may be due to the high-risk perception of the virus and high testing and vaccination capacity during this time but is likely to differ in a routine context. The descriptive results of this study are likely to be useful to clinicians and policy makers interested in the characteristics of individuals who are more likely to take up vaccinations and other nationwide programs. We showed that people who take up UK nationwide screening programs and NHS health checks are more likely to get vaccinated. Policy makers could use this information to improve health equity.

Our study was strengthened by the large cohort and harmonized analyses with consistent variable definitions and modeling approaches before and during the COVID-19 pandemic. Some previous VE analyses have adjusted for single variables that aim to capture health-seeking behavior [8, 9, 12]. We included a set of proxy markers based on a theoretical model [13], providing a more systematic approach to adjusting for this complex phenomenon that can be used in other observational studies with routinely collected data. Although other approaches (eg, instrumental variables) can be used to address bias from unobserved confounders [39], our multivariate modeling approach enabled us to quantify and control for the confounding captured by the included markers. We were also able to identify and quantify residual confounding using a negative control exposure and demonstrate the impact of adjusting for health-seeking behavior and health care access.

Despite these strengths, limitations remain. We assessed health-seeking behavior at the index date, but this might change over time, especially for the COVID-19 analysis, in which risk perception likely influenced health behaviors. There could be other time-varying confounding [40] if, for example, nonvaccination leads to infection and temporary ineligibility for vaccination [41]. There is scope for selection bias in the negative control exposure analysis; if individuals vaccinated against influenza from 2018 to 2019 were less likely to die in the interim before the start of follow-up for COVID-19 in January 2020, then this could overestimate VE slightly. In the future, it would be useful to understand how these markers perform (together, individually, or in various subsets) and adapt to different age groups, settings, databases, study types, and research questions, including designs that explicitly account for time-varying confounders [41]. Specifically, it would be beneficial to apply the same analytic framework to study the incremental effectiveness of more recent COVID-19 booster campaigns, given potential changes in health-seeking behavior as we emerge from the pandemic.

## CONCLUSION

We have identified markers in UK EHRs that can be used to quantify and adjust for confounding from health-seeking behavior and health care access in observational research. Adjusting for health-seeking behavior had a limited influence on estimates of COVID-19 VE during the pandemic early vaccine rollout. For seasonal influenza VE, severe outcomes were robust to confounding from health-seeking behavior, but VE against ILI was underestimated prior to adjustment for health-seeking behavior. Residual confounding was also removed, as demonstrated in a negative control exposure analysis of history of influenza vaccination against COVID-19 infections.

## Supplementary Data

Supplementary materials are available at *Open Forum Infectious Diseases* online. Consisting of data provided by the authors to benefit the reader, the posted materials are not copyedited and are the sole responsibility of the authors, so questions or comments should be addressed to the corresponding author.

## Supplementary Material

ofae598_Supplementary_Data

## Notes

***Acknowledgments.*** This work uses data provided by patients and collected by the NHS as part of their care and support (usemydata.org).

***Data availability statement.*** These data were obtained from the CPRD, provided by the UK Medicines and Healthcare products Regulatory Agency. The authors' licence for using these data does not allow sharing of raw data with third parties. Information about access to CPRD data is available here: https://www.cprd.com/research-applications.

***Disclaimer.*** The views expressed are those of the authors and not necessarily those of the NHS, National Institute of Health and Care Research, Department of Health and Social Care, or UK Health Security Agency.

***Ethics and CPRD requirements.*** We received data governance approval from CPRD (protocol 21_000737) and ethics approval from London School of Hygiene and Tropical Medicine’s independent ethics committee (28169).

***Open science.*** All the analyses were conducted with R versions 4.2.2 to 4.2.4. All programming code from this project can be found in the GitHub repository: https://github.com/grahams99/Health-seeking-behaviour. The code lists and related search terms can be found on the London School of Hygiene and Tropical Medicine’s Data Compass: https://doi.org/10.17037/DATA.00003684.

***Financial support.*** This work was supported by the National Institute of Health and Care Research’s Health Protection Research Unit in Vaccines and Immunisation (grant NIHR200929 to S. G., E. P. K. P., N. A., J. L. W., and H. I. M.), a partnership between the UK Health Security Agency and London School of Hygiene and Tropical Medicine.
